# Health benefits of dancing activity among Korean middle-aged women

**DOI:** 10.3402/qhw.v11.31215

**Published:** 2016-07-04

**Authors:** Min Jeong Kim, Chul Won Lee

**Affiliations:** 1 Department of Global Sport, Hankuk University of Foreign Studies, Seoul, South Korea; 2 Department of Sport and Leisure Studies, Yonsei University, Seoul, South Korea

**Keywords:** Health benefit, leisure experience, Korean middle-aged women, line dancing, serious leisure

## Abstract

The purpose of this study was to understand the health benefits of line dancing activity in Korean middle-aged women. This study explored how Korean middle-aged women perceive health benefits through lived experiences of line dancing in their leisure time. Three themes emerged related to health benefits: (1) psychological benefit, (2) physical benefit, and (3) social benefit. This finding suggested that serious leisure experience aids health enhancements in the lives of Korean middle-aged women. This study also discusses the research implication that continuous participation in leisure activity is necessary for health improvement in Korean middle-aged women.

In Korean society, middle-aged women are a vulnerable class from the perspective of health. Physiologically, they experience the onset of aging, and mentally, they experience strong feelings of conflict and a sense of crisis. In the family, their children start to be independent and, due to the retirement and unemployment of their husbands, the women start to become sensitive to economic crisis. During this phase, these women start to have concerns regarding their own identity. According to 2012 statistics, the rate of death due to hypertensive disease among women was 2.25 times that among men, and the percentage of women who felt the urge to commit suicide was 10.6%, 7.5% higher than that of men. The reasons for considering suicide among middle-aged women especially were economic troubles (35.2%), family discord (15.1%), and loneliness (14.9%) (Statistics Korea, 2014).

Physiological changes are the most tangible changes experienced by women. Through menopause, ovary function declines dramatically, and estrogen levels decrease, which causes a weakening of physical function. Such physiological change induces osteoporosis and cardiovascular disorders. The problem is that such physiological change also induces mental problems. The sense of chaos that results from the change in roles, the conflict that results from the independence of their children, the upsurge of dejection, and the depression and bipolar disorder that emerge without cause threatens women's mental health (Park & Kim, 2010). However, this period is not always negative. Through this period, women adapt emotionally, socially, and culturally. Adequate leisure experiences during this period especially have a static influence on satisfaction in life and the enhancement of happiness.

Previous studies have determined that middle-aged women have a difficult time social-psychologically. In this period, middle-aged women may be prone to anger that, if it not resolved successfully, may led to diseases such as migraines and obesity (Russell & Shrink, 1993). If this period is overcome successfully, self-respect increases and these women may form a healthy ego. Additionally, they may reach old age healthily. A great deal of research has been conducted in the past on the effects of exercise in menopausal women. Many studies have pointed out the importance of exercise in preventing physiological health diseases (Gordon, Overend, & Vandervoort, 2001; McKinlay, McKinlay, & Brambilla, 1987), but research is currently lacking that suggests effects of leisure activities as methods to overcome the mental plight that can be experienced after menopause.

Although Korean middle-aged women experience health benefits through meaningful leisure activities, detailed research on what leisure activities grant health benefits is also lacking. Therefore, this research intends to understand the health benefits of middle-aged women who participate in line dancing.

## Serious leisure experience and health promotion

Leisure activities in adults help to enhance physical and mental health (Sener, Terzioglu, & Karabulut, 2007; Yau & Packer, 2002). The physical and mental health state that has been enhanced through leisure activities also affects satisfaction in life or improvement in happiness. Physically active leisure in middle age especially enhances mental health (Kim, Yamada, Heo, & Han, 2014; Stathi, Fox, & McKenna, 2002).

Previous studies have proposed that physical activity brings various health benefits to middle-aged women. Physical activity especially contributes to improvement in their mental health (Stathi et al., 2002). According to Slaven and Lee (1997), continuous participation by middle-aged women in exercise changes mood and helps them to solve the psychological problems resulting from menopause. Due to menopause, middle-aged women become weaker physically and discouraged psychologically. According to Parry and Shaw (1999), physically active leisure contributes to heightened physical and emotional well-being among middle-aged women. Physical leisure activities have a decisive role in maintaining physical and mental health in life after middle age (Dionigi, 2006; Kim, Kim, Han, & Chin, 2015; Lawton, 1994).

Those who participate seriously in leisure activities experience high levels of satisfaction in life. Stebbins (1992) suggested that this follows the systematic leisure experiences of amateur, hobbyist, or volunteer behavior related to substantial and interesting leisure participation. In this context, Stebbins (2001) has proposed six characteristics to separate routine leisure from serious leisure: perseverance, career, personal effort, durable benefits, rewards, strong identity, and unique ethos.

For serious leisure participants after middle age, especially, the purpose of participating in leisure activities has a deep relation to the pursuit of happiness (Brown, McGuire, & Voelkl, 2008). According to Siegenthaler and O'Dell (2003), older adults who seriously participate in golf experience improved well-being as a result. Therefore, serious participation in leisure activities after one's middle years contributes to increases in life satisfaction and well-being. According to Cheng (2010), in the research on how the level of participation, serious leisure, hobby leisure, and casual leisure affect life satisfaction, serious leisure participants have felt the highest amount of life satisfaction. Serious participation in leisure activities helps to improve mental health and enhances happiness.

Previous studies have shown that the participation in leisure among middle-aged women helps to promote their health (Choi, 2006; Kim et al., 2015; Park & Kim, 2010). In this research, there are two reasons for selecting Korean middle-aged women who participate in line dancing as research participants. First, in Korean society, middle-aged women are recognized as the most vulnerable class regarding health. Second, there is no research whatsoever regarding the health benefits to Korean middle-aged women who participate seriously in leisure activities. From this point of view, this research will verify the role of serious participation in line dancing in maintaining health among Korean middle-aged women.

## Methods

This research intends to include a phenomenological analysis of the leisure benefits recognized by middle-aged women through line dance participation. Applying Van Mannen's (1990) research on analytical phenomenology, we intended to describe the meaning of experience for the research participants “as is.”

The research method of analytical phenomenology has a few characteristics. First, phenomenological research researches the experience. Second, it explains the phenomenon as it appears on the consciousness. Third, it researches the essence. Fourth, it narrates the meaning of experience as we experience it. Fifth, it is human scientific research into the phenomenon. Sixth, it is a cautious practice of thoughtfulness. Seventh, it researches what it means to be humane. Therefore, with these phenomenological characteristics as a basis, the researchers attempted to understand the health benefits that result from serious participation in leisure activities in middle-aged women's lives.

### Selecting participants and ethical consideration

As the participants for this research, we selected eight middle-aged women who were seriously participating in line dancing. Line dancing means the dancing style with a repeated steps where people dance in various lines facing all people in the same direction, and performing specific steps (Wikipedia, 2016). The participants were participating in line dance lessons at the extension of Seoul's Yonsei University, and their ages ranged from 41 to 65. The participants were introduced to line dancing by a friend's suggestion, introduction to the dance teacher, other advertisement data, and various other avenues, and they had started line dancing between 1 and 3 years previously (Table I).

**Table I:** Participant characteristics.

Name	Age	Line dancing career (years)
Song	47	3
Kim	45	4
Choi	57	3
Ku	45	4
Chon	65	4
Young	41	3
Won	45	1
Yoon	53	1

The purposive sampling method was applied for the standard of selection for the participants. At the extension of the university, the line dance instructor and the researchers questioned the participants on their experience. In the end, three researchers were sent to the site to collect and analyze data.

For moral considerations regarding the participants, the researcher primarily received a simple written response. Through this written response, the researchers promised that the information provided by the participants would not be used other than for the purpose of research and that the participants would remain anonymous. In the data-collecting procedure, the questions were recorded, and the researchers received permission to use them for the study.

### Data collection and analysis

The main data collection of this research was conducted through in-depth interviews. The areas that require careful consideration by the researchers in phenomenology are categorized by phenomenon. Accordingly, researchers first assessed the prejudices regarding middle-aged women's physiological and mental problems and organized the researchers’ preconceptions and prior knowledge. Afterwards, excluding the magnified prejudices, the researcher questioned the participants using an open-ended question on the life of a middle-aged woman. After that, the researcher questioned the participants on the meaning of middle age, change, and the meaning and advantages of line dancing as a serious leisure activity.

The in-depth interviews took place after the participants’ line dance lessons in the classroom or the laboratory of the researcher. The interviews were less than 1 h per session, and after the first interview, if needed, additional questions were asked through a second interview or phone call. The in-depth interview data were recorded with a cellular phone and transcribed. The data were collected from March 2010 to April 2011.

This research was based on the phenomenological research proposed by Van Mannen (1990) to analyze the data. The researcher, attempting to understand the meaning of the experience of the participants, has accommodated a “researcher as an instrument” (Morse & Field, 1995, p. 141) point of view.

### Trustworthiness

To secure the accuracy of the data through member check, the research participants were asked to confirm the research data. When collecting and analyzing the data, the triangulation method was applied. Peer debriefing to verify the validity of the research determined that the interpretative validity, relatedness to the research problem, and the appropriateness of the analyzing method were to be increased (Lincoln & Guba, 1999).

## Findings

Middle-aged women find joy in leisure activities they have chosen themselves, and they were aware of various health benefits of participation in line dancing. With their serious leisure experience of line dancing as a background, various health benefits were stated in detail. The research participants mentioned psychological, physical, and social benefits.

### Psychological benefit

Every participant mentioned the psychological benefits of line dancing often. The participants often experienced psychological anxiety in their daily lives. Depression, especially through middle age, is a dangerous psychological pathological phenomenon (Park & Kim, 2010). In relieving such psychological anxiety, the participants stated that line dance participation had a good effect.

The participants said that, through line dancing, they had time to recognize their psychological well-being. They expressed that, since starting line dancing, they have become able to live with the recognition of what well-being is: “For now, by starting line dancing, I started to feel my well-being and to think it is good for my mental health and will help with preventing dementia in the future” (Kim, 45).

Most participants were very sensitive regarding “mental health.” Most had experienced depression and said that depression was the crisis of their lives. Choi (57) described the feeling experienced when the depression first came as “isolation.” Chon (65) said that she experienced a strong sense of psychological well-being while line dancing that she did not feel during other leisure activities. She said in particular that she has noted a feeling of growing younger while participating in line dancing. She had a regular retirement as an elementary school teacher 3 years ago. Afterward, she tried art and tried to learn Korean dancing. She said that her life changed when she was introduced to line dancing. She started to think that she is growing younger by dancing along with music, and this change heightened her psychological well-being. Song (47), who had a difficult time with depression, mentioned the following:

For people in my age group, children go to school and come back late, and as the school lunches are done well, they come back late after eating. In such an environment, in order to avoid depression, which our age group is prone to, by doing adequate amounts of leisure activities, I think that leisure activities such as line dancing are necessary. In addition, in order to be satisfied with one's own life, I think line dance participation is necessary.

For the participants, the experience of line dancing was “a road to myself.” As housewives and mothers of children, they had no opportunity to look after their leisure. After middle age, once they had abundant leisure time, they were able to think deeply on their leisure, and they were able to self-examine their lives at the same time.

Some say that middle age is a time of “loneliness.” The main cause of such loneliness, according to Choi (57), is the increase in anxiety as men have settled down, to a certain degree, outside and children are growing up. In a psychologically unstable period, middle-aged women have an opportunity to reflect on their leisure and lives. It was shown that children were satisfied that their mothers spent their leisure time splendidly by line dancing. In this aspect, according to Song (47), children prefer a mother who performs leisure activities outside rather than one who always stays at home. She said that this is because children feel that the fact that their mothers only stay at home, while they grow up and have their own lives, is a pity. Kim (45) also said that the children dislike a mother who stays at home and prefer a mother who immerses herself in line dancing. To the participants, line dancing is becoming a medium and experience that enriches their lives.

Middle-aged women are excessively exposed to stress. Song (47) and Choi (57) also emphasized that line dance experience has a positive effect in relieving stress. “Thinking of line dancing is always a joyful thing,” they said. They also said that line dancing is effective in stress prevention.

### Physical benefit

Physical benefits, along with psychological benefits, are one of the visible changes through line dancing. Song (47) said that the exercise has a positive effect on the heart and the cardiovascular system:

You can feel the clear difference if you do it after not exercising. Just by doing line dance one hour per day, I think it has an equivalent effect of walking 7000–10000 steps. If one exercises every day, then I believe it will have a great exercise effect.

The participants said that, by realizing that their bodies will become healthy by line dancing, they fall for the charms of line dancing.

The advantages of line dancing are that, although it is not difficult as it is done along with the music, the amount of exercise done is considerable. Most participants became aware of health problems as they entered middle age. They said that they realized that they became healthier through line dancing. Choi (57) said the following about the physical strengths of line dancing: “As I don't overdo it physically and come in contact with music in an easy way, it is not boring. Not overdoing it physically and not being boring is, I think, the greatest strength of line dancing.”

Choi (57), Young (41), and Won (45) reported that, because their legs were weak, they were not able to exercise for long in the past. They said that, as they aged, their legs weakened first. They said in unison that, in order to overcome a weakened lower body, line dancing is effective. By performing aerobic exercise regularly, they reported that they saw such effects as prevention of cardiovascular disorders, the eradication of arthritis, strengthening of the lower muscles, and other health benefits.

Ku (45) reported that the biggest effect of line dancing was “muscle increase”:

I think it would be nice for increasing muscular strength. I had weak legs, so I tried mountain climbing as well, but I had a difficult time. It was difficult when climbing up and also difficult when descending back down. However, after doing line dance, I couldn't feel difficulty even after walking a lot.

The participants also said that they received a great deal of help with “fat reduction” while doing line dance. Choi (57) said that, by continuing exercise, she felt that her stamina had increased and that she had become more fit. As line dancing is an aerobic exercise, when performed for over 30 min, it aids considerably with weight loss. The problem with aerobic exercise is participating continuously, and in order to continuously participate, it has to be “fun.” Line dancing, of course, is fun as it is performed while listening to music, so it is a great deal of help in fitness.

Middle-aged women are vulnerable to obesity. The reason for their obesity is the lack of exercise and poor eating patterns. Generally, the reason they lack exercise is that, during their leisure time, which has suddenly increased, they do not participate in exercise as they lack exercise skills. Song (47) said that line dancing involves a considerable amount of exercise. The reason line dancing is good for obesity prevention, especially, is because, along with music, physical activity is performed so it has good dieting effects.

### Social benefit

Through line dance activities, the participants have become very close to each other. By exercising and sweating together, they said that they have felt close. To women, middle age is a lonely time. By conversation with others living in this period of time together, they were able to feel a sense of kinship. Line dancing lessons provided a place where they shared the same worries and could talk about them. Most participants said that, through line dance meetings, they were able to feel strong social closeness. After middle age, it is difficult to make friends, but through line dance meetings, these women were able to make new friends and meet with old friends. In this way, line dance participation is a meaningful way to recover closeness between friends, an experience that the participants felt was mostly in the past, and had been lost.

Song (47) said, “Friendship has been strengthened as people who are interested in the same exercise met together.” To middle-aged women, line dancing is not a mere exercise; it is also a leisure activity that forms social relationships. Chon (65) was able to become aware of many changes by doing line dance, and she tries to encourage others to participate in good exercise as well. She volunteers in teaching many grandmothers in the town. She said that, as she herself has become psychologically stable and physically healthy, she gained the will to volunteer to help others. She said that she gained the will through line dancing to volunteer and share the good feelings she has realized with others.

Kim (45) said that she is learning to share “something” through line dancing. She was so immersed in line dancing that she has become a line dance instructor herself. Although she practices line dancing in her leisure time as well, she said that she usually gains satisfaction through the act of teaching others. The important benefit of line dance participation is the formation of the concept of social leisure, where one wishes to share one's own private joys with others.

Most research participants experienced a great deal of stress due to their children's studies. For the method to relieve such stress, they have said that meeting other people and sharing their experiences and having conversation was effective. Ku (45) said that her eldest child had too much stress from her last year of high school the previous year, so she had great concerns as a mother. Her eldest child apparently was rebellious from the first year of middle school to the last year of high school, and was difficult to raise. During that time, when Ku had a hard time in various difficult situations, after exercising, she would talk out her dilemmas with line-dancing friends who helped her work out her problems. The participants resolved psychological difficulties through line dancing and also strengthened their social abilities.

## Discussion

In Korean society, interest in health benefits through leisure activities in middle-aged women is increasing. Middle-aged women realize the psychological, physical, and social benefits of line dancing. Such health benefits have been revealed to have an important role in increasing their happiness.

According to previous research, psychological and physical weakening starts after menopause in particular (McKinlay et al., 1987; Parry & Shaw, 1999; Sener, Terzioglu, and Karabulut 2007). As menopause continues, physical abilities start to decrease, and the onset of osteoporosis and decreases in cardiovascular function are experienced. Other than that, middle-aged women experience psychological, pathological phenomena due to the decline of their husbands’ economic ability, the independence of their children, and the upsurge of feelings of loss. According to Park and Kim (2010), Korean women go through serious intimidation in various mental health issues in menopause, and to relieve that, an adequate amount of physical activity is necessary. In this research as well, the participants were experiencing serious stress, but through line dancing, they found a method to relieve stress (Nadasen, 2008).

According to previous studies, middle-aged women are in a vulnerable zone of health (Freysinger, 1995; Park & Choi, 2007; Slaven & Lee, 1997). According to Park and Choi (2007), Korean middle-aged women, in order to overcome the “crisis” of life, develop a new awareness of health management. In this research as well, a few participants had experienced depression and life crises, which they were able to wisely overcome through the activity of line dancing. Additionally, in their crises, through leisure activities, they pursued problem-solving by conversing with other middle-aged women friends.

The research results in leisure studies have reported that adequate amounts of leisure activities in the lives of middle-aged women give them physical, psychological, and social benefits (Choi, 2006; Kim et al., 2015; Parry & Shaw, 1999; Son, Kim, & Kim, 2005). Serious leisure participation after middle age especially has an important role in enhancing health physically, psychologically, and socially (Cheng, 2010; Lawton, 1994; Siegenthaler & O'Dell, 2003; Stathi et al., 2002). From this perspective, according to Son et al. (2005), an activity that can maintain the balance of body and heart in the lives of middle-aged women can maintain health. In this study, participants were shown, through line dancing, how to maintain their health through the balanced change in body and heart.

After middle age, women are exposed to the problem of obesity as they lack exercise. Continuous participation in line dancing reduces fat and helps prevent obesity. The problem in Korean middle-aged housewives’ leisure participation lies in the unclear border between leisure and non-leisure. To them, their leisure is not a priority compared to other things. Due to this problem, the participants in this research also could not participate in leisure activities regularly, which had a big influence in obesity. At that time, line dance participation was very effective in eliminating obesity.

Through this research, it has been suggested that serious leisure experience helps to aid health enhancements in the lives of Korean middle-aged women (Kim, Heo, & Kim, 2014). Dance experiences, like line dancing especially, provide middle-aged women with psychological, physical, and social benefits. Unlike other leisure activities, line dancing is fun and is an activity in which it is possible to become immersed. In order to improve Korean middle-aged women's health, this research has shown that it is important to induce participation in a leisure activity that they may be interested in, such as line dancing.

### Limitations and need for future research

Three proposals are supported by this research. First, this research aimed to interpret the benefits of serious line dance experience in middle-aged women through qualitative research. Further research is necessary to analyze the experiences of middle-aged women who seriously participate in activities other than line dancing, such as swimming and yoga. Research into the different health benefits of leisure activities preferred by Korean middle-aged women also seems necessary.

Second, vertical research is needed on the health benefits of middle-aged women's leisure participation. Interpretations of how the health benefits shown in this research have changed and strengthened as the term of line dance participation increases are also necessary and must be obtained through long-term research.

Third, an analysis of how awareness of health benefits through the leisure participation of Korean middle-aged women affects their satisfaction in life or happiness is necessary. Physical and psychological health influences successful aging (Brown et al., 2008).

In conclusion, this research suggested that the leisure activity of line dancing provides health benefits in the lives of Korean middle-aged women. The meaning of this lies in the suggestion that continuous participation in a leisure activity is necessary for the improvement of health.
